# 4,4′-Dichloro-3,3′,5,5′-tetra­methyl-2,2′-[(3a*R*,7a*R*/3a*S*,7a*S*)-2,3,3a,4,5,6,7,7a-octa­hydro-1*H*-1,3-benzimidazole-1,3-di­yl)bis­(methyl­ene)]diphenol

**DOI:** 10.1107/S1600536811028960

**Published:** 2011-07-23

**Authors:** Augusto Rivera, Diego Quiroga, Jaime Ríos-Motta, Karla Fejfarová, Michal Dušek

**Affiliations:** a Departamento de Química, Universidad Nacional de Colombia, Ciudad, Universitaria, Bogotá, Colombia; b Institute of Physics ASCR, v.v.i., Na Slovance 2, 182 21 Praha 8, Czech Republic

## Abstract

In the title compound, C_25_H_32_Cl_2_N_2_O_2_, there are two intra­molecular O—H⋯ N hydrogen-bonding inter­actions between the hy­droxy groups on the aromatic rings and the two N atoms of the heterocyclic group. The cyclo­hexane ring adopts a chair conformation and the imidazolidine unit to which it is fused has a twisted envelope conformation. The asymmetric unit comprises one half-mol­ecule which is completed by a twofold rotation axis. A C—H⋯O inter­action is observed in the crystal structure.

## Related literature

For related structures, see: Rivera *et al.* (2010 ▶); Cox (1995 ▶). For related quantum-chemical literature, see: Zierkiewicz *et al.* (2000 ▶, 2003 ▶, 2004 ▶).
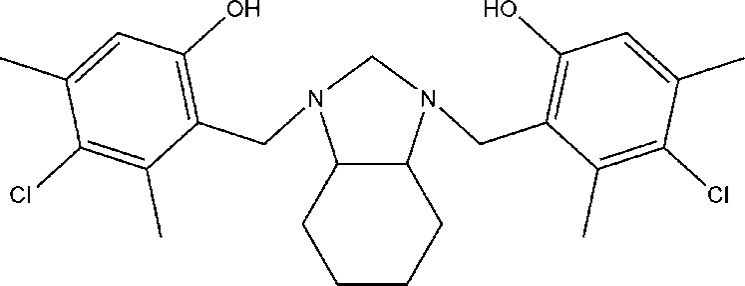


## Experimental

### 

#### Crystal data

C_25_H_32_Cl_2_N_2_O_2_
*M* *_r_* = 463.4
Monoclinic, 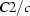

*a* = 16.6512 (7) Å
*b* = 9.6962 (3) Å
*c* = 14.4423 (6) Å
β = 98.892 (3)°
*V* = 2303.73 (15) Å^3^
*Z* = 4
Cu *K*α radiation
μ = 2.73 mm^−1^
*T* = 120 K
0.53 × 0.36 × 0.16 mm

#### Data collection

Agilent Xcalibur diffractometer with Atlas Gemini detector
Absorption correction: analytical (*CrysAlis PRO*; Agilent, 2010 ▶)’ *T*_min_ = 0.411, *T*_max_ = 0.734
32618 measured reflections
2054 independent reflections
1979 reflections with *I* > 3σ(*I*)
*R*_int_ = 0.046

#### Refinement

*R*[*F* ^2^ > 2σ(*F* ^2^)] = 0.033
*wR*(*F* ^2^) = 0.116
*S* = 2.54
2054 reflections
144 parameters
1 restraint
H atoms treated by a mixture of independent and constrained refinement
Δρ_max_ = 0.27 e Å^−3^
Δρ_min_ = −0.24 e Å^−3^

### 

Data collection: *CrysAlis PRO* (Agilent, 2010 ▶); cell refinement: *CrysAlis PRO*; data reduction: *CrysAlis PRO*; program(s) used to solve structure: *SIR2002* (Burla *et al.*, 2003 ▶); program(s) used to refine structure: *Jana2006* (Petříček *et al.*, 2006 ▶); molecular graphics: Diamond (Brandenburg & Putz, 2005 ▶); software used to prepare material for publication: *Jana2006*.

## Supplementary Material

Crystal structure: contains datablock(s) global, I. DOI: 10.1107/S1600536811028960/go2020sup1.cif

Structure factors: contains datablock(s) I. DOI: 10.1107/S1600536811028960/go2020Isup2.hkl

Additional supplementary materials:  crystallographic information; 3D view; checkCIF report

## Figures and Tables

**Table 1:** Hydrogen-bond geometry (Å, °)

*D*—H⋯*A*	*D*—H	H⋯*A*	*D*⋯*A*	*D*—H⋯*A*
O1—H1⋯N1	0.827 (17)	1.880 (19)	2.6259 (13)	149.4 (19)
C12—H12B⋯O1^i^	0.96	2.56	3.4998 (17)	166

Symmetry code: (i) 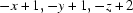.

**Fig. 1.**
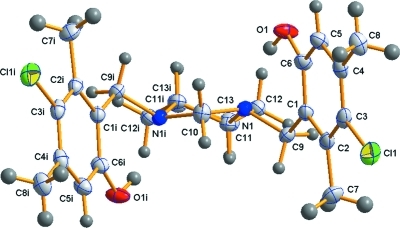
A view of (I) with the numbering scheme. Displacement ellipsoids are drawn at the 50% probability level. Symmetry codes: i(-x, y, -z+1/2).

## supplementary crystallographic information

### Comment

The presence of *p*-halo substituent in the phenol ring afforded structural consequences such as the deformation of the ring observable in the bond distances and the bond angles values, which is related with the existence of resonance effect (*X* = Br, Cl) and inductive effect (*X* = F), according to theoretical results using the MP2 and density functional (B3LYP) methods (Zierkiewicz, *et al.* 2000 and 2003). Theoretical investigations using NBO analysis suggested that *p*-chloro substituent induces a decrease of electron density in the lone pair orbital of the O atom with a reinforcement of the delocalization of electronic density to aromatic ring observable in a slight shortening of C—O and C—C bonds (Zierkiewicz, *et al.* 2004). With the aim to understand the effect of electron-donating groups in the *p*-halophenol derivatives, we synthesized the title compound (I).

The molecular structure and atom-numbering scheme for (I) are shown in Fig. 1. Selected angles and bond lengths are listed in Table 1. These results show the existence of intramolecular hydrogen bonding interactions between the hydroxy H atom and the nitrogen atoms in the imidazolidine moiety. The shorter H—O distance (0.827 (17) Å) in comparison with the *p*-chlorophenol derivative (Rivera, *et al.* 2010), indicates a decreasing hydrogen-bonding strength. However, since the N···H and the N···O distances (table 1) are longer by 0.05 Å and 0.03 Å and the observed C—O bond length (1.3612 (17) Å) is in a good agreement with the mentioned related structure, we concluded that the methyl groups do not induce considerably the decrease in hydrogen-bonding strength despite the electron-donating effect on the aromatic rings.

However, the observed C1—C2 and C4—C5 bond length are longer in comparison with the 3,5-dimethyl-4-chlorophenol (Cox, 1995) and the *p*-chlorophenol derivative (Rivera, *et al.* 2010), indicating a lower tendency to form a quinoid-type structure, reducing the delocalization of electronic density presumably due the electron-donating effect of the methyl groups in the 3 and 5 positions.

### Experimental

A solution of 3,5-dimethyl-4-chlorophenol (313 mg, 2.00 mmol) in dioxane (3 ml) was added dropwise to a solution of (2*R*,7*R*,11*S*,16S)-1,8,10,17-tetraazapentacyclo [8.8.1.1^8,17^.0^2,7^.0^11,16^]icosane (276 mg, 1.00 mmol) prepared beforehand following previously described procedures, in dioxane (3 ml) and water (4 ml). The mixture was refluxed for about 8 h until precipitation of a colourless solid. The resulting solid was collected by filtration, washed with cool methanol and dried under vacuum (yield 50%, m.p. = 497–499 K). Single crystals of racemic (I) were grown from a CHCl_3_ solution by slow evaporation of the solvent at room temperature over a period of about 2 weeks.

### Refinement

All hydrogen atoms were discernible in difference Fourier maps and could be refined to reasonable geometry. According to common practice they were nevertheless kept in ideal positions with C–H distance 0.96 Å during the refinement. The methyl H atoms were allowed to rotate freely about the adjacent C—C bonds. The hydroxyl hydrogen atom was refined with a distance restraint d(O—H) = 0.84 (2) Å. The isotropic atomic displacement parameters of hydrogen atoms were evaluated as 1.2–1.5×*U*_eq_ of the parent atom.

### Crystal data

**Table:**

C_25_H_32_Cl_2_N_2_O_2_	*F*(000) = 984
*M**_r_* = 463.4	*D*_x_ = 1.336 Mg m^−^^3^
Monoclinic, *C*2/*c*	Melting point: 498 K
Hall symbol: -C 2yc	Cu *K*α radiation, λ = 1.5418 Å
*a* = 16.6512 (7) Å	Cell parameters from 23197 reflections
*b* = 9.6962 (3) Å	θ = 3.1–67°
*c* = 14.4423 (6) Å	µ = 2.73 mm^−^^1^
β = 98.892 (3)°	*T* = 120 K
*V* = 2303.73 (15) Å^3^	Block, colourless
*Z* = 4	0.53 × 0.36 × 0.16 mm

### Data collection

**Table:**

Agilent Xcalibur diffractometer with Atlas Gemini detector	2054 independent reflections
Radiation source: Enhance Ultra (Cu) X-ray Source	1979 reflections with *I* > 3σ(*I*)
mirror	*R*_int_ = 0.046
Detector resolution: 10.3784 pixels mm^-1^	θ_max_ = 67.2°, θ_min_ = 5.3°
Rotation method data acquisition using ω scans	*h* = −19→19
Absorption correction: analytical (*CrysAlis PRO*; Agilent, 2010)'	*k* = −11→11
*T*_min_ = 0.411, *T*_max_ = 0.734	*l* = −17→17
32618 measured reflections	

### Refinement

**Table:**

Refinement on *F*^2^	61 constraints
*R*[*F*^2^ > 2σ(*F*^2^)] = 0.033	H atoms treated by a mixture of independent and constrained refinement
*wR*(*F*^2^) = 0.116	Weighting scheme based on measured s.u.'s *w* = 1/(σ^2^(*I*) + 0.0016*I*^2^)
*S* = 2.54	(Δ/σ)_max_ = 0.010
2054 reflections	Δρ_max_ = 0.27 e Å^−^^3^
144 parameters	Δρ_min_ = −0.24 e Å^−^^3^
1 restraint	

### Special details

**Table:**

Experimental. ^1^H NMR (CDCl3, 400 MHz): δ 1.31 (4H, m), 1.87 (2H, m), 2.08 (2H, m), 2.26 (2H, s, ArCH3), 2.28 (2H, s, ArCH3), 2.36 (2H, m), 2.46 (2H, s, NCH2N), 3.68 (2H, d, 2J = 14.0 Hz, ArCH2N), 4.05 (2H, d, 2J = 14.0 Hz, ArCH2N), 6.57 (2H, s), 11.18 (2H, bs). 13C NMR (CDCl3, 100 MHz): δ 16.7, 21.0, 24.0, 28.9, 53.0, 69.2, 75.8, 116.4, 118.3, 125.4, 133.5, 136.7, 155.9.
Refinement. The refinement was carried out against all reflections. The conventional *R*-factor is always based on *F*. The goodness of fit as well as the weighted *R*-factor are based on *F* and *F*^2^ for refinement carried out on *F* and *F*^2^, respectively. The threshold expression is used only for calculating *R*-factors *etc*. and it is not relevant to the choice of reflections for refinement.The program used for refinement, Jana2006, uses the weighting scheme based on the experimental expectations, see _refine_ls_weighting_details, that does not force *S* to be one. Therefore the values of *S* are usually larger than the ones from the *SHELX* program.

### Fractional atomic coordinates and isotropic or equivalent isotropic displacement parameters (Å^2^)

**Table:**

	*x*	*y*	*z*	*U*_iso_*/*U*_eq_	
Cl1	0.76734 (2)	−0.07704 (4)	0.93216 (3)	0.03586 (17)	
O1	0.53003 (6)	0.35475 (10)	0.97680 (7)	0.0322 (3)	
N1	0.55223 (4)	0.45810 (9)	0.81461 (5)	0.0204 (3)	
C1	0.63836 (7)	0.27435 (13)	0.89563 (9)	0.0222 (4)	
C2	0.69590 (8)	0.17091 (14)	0.88695 (9)	0.0241 (4)	
C3	0.69590 (8)	0.05287 (14)	0.94211 (9)	0.0258 (4)	
C4	0.64130 (8)	0.03133 (14)	1.00472 (9)	0.0261 (4)	
C5	0.58507 (9)	0.13507 (14)	1.01187 (9)	0.0264 (4)	
C6	0.58435 (8)	0.25638 (14)	0.96017 (9)	0.0247 (4)	
C7	0.75806 (9)	0.18747 (17)	0.82207 (11)	0.0348 (5)	
C8	0.64127 (10)	−0.09641 (15)	1.06364 (10)	0.0326 (4)	
C9	0.63587 (8)	0.40679 (14)	0.83881 (9)	0.0237 (4)	
C10	0.5	0.36595 (10)	0.75	0.0261 (5)	
C11	0.54490 (7)	0.59416 (13)	0.76934 (8)	0.0197 (4)	
C12	0.56829 (8)	0.71792 (13)	0.83144 (9)	0.0231 (4)	
C13	0.54453 (8)	0.84894 (13)	0.77370 (9)	0.0267 (4)	
H1	0.5257 (13)	0.4090 (18)	0.9321 (12)	0.0387*	
H5	0.545829	0.122861	1.053384	0.0317*	
H7a	0.810554	0.160357	0.854033	0.0522*	
H7b	0.743354	0.130427	0.767765	0.0522*	
H7c	0.759828	0.28218	0.803107	0.0522*	
H8a	0.694459	−0.110125	1.098872	0.0488*	
H8b	0.6025	−0.08608	1.105925	0.0488*	
H8c	0.626841	−0.174614	1.023772	0.0488*	
H9a	0.668777	0.475635	0.874286	0.0284*	
H9b	0.658249	0.390095	0.782437	0.0284*	
H10	0.533179	0.311486	0.715257	0.0313*	
H11	0.582025	0.604471	0.725135	0.0237*	
H12a	0.625937	0.717436	0.852045	0.0277*	
H12b	0.539438	0.71524	0.884052	0.0277*	
H13a	0.57933	0.859112	0.726983	0.032*	
H13b	0.554553	0.928472	0.813392	0.032*	

### Atomic displacement parameters (Å^2^)

**Table:**

	*U*^11^	*U*^22^	*U*^33^	*U*^12^	*U*^13^	*U*^23^
Cl1	0.0336 (3)	0.0301 (3)	0.0424 (3)	0.01390 (13)	0.00143 (18)	−0.00539 (13)
O1	0.0418 (6)	0.0280 (5)	0.0313 (5)	0.0149 (4)	0.0196 (4)	0.0086 (4)
N1	0.0172 (5)	0.0215 (5)	0.0224 (5)	0.0011 (4)	0.0029 (4)	0.0007 (4)
C1	0.0201 (6)	0.0247 (7)	0.0209 (6)	0.0021 (5)	0.0003 (5)	−0.0019 (5)
C2	0.0202 (6)	0.0272 (7)	0.0235 (6)	0.0018 (5)	−0.0006 (5)	−0.0065 (5)
C3	0.0231 (7)	0.0245 (6)	0.0274 (7)	0.0061 (5)	−0.0035 (5)	−0.0075 (5)
C4	0.0307 (7)	0.0235 (7)	0.0217 (6)	0.0032 (5)	−0.0032 (5)	−0.0050 (5)
C5	0.0321 (7)	0.0258 (7)	0.0218 (6)	0.0046 (5)	0.0058 (5)	0.0003 (5)
C6	0.0271 (7)	0.0258 (6)	0.0217 (6)	0.0056 (5)	0.0049 (5)	−0.0019 (5)
C7	0.0263 (7)	0.0401 (8)	0.0393 (8)	0.0072 (6)	0.0096 (6)	−0.0021 (6)
C8	0.0404 (8)	0.0252 (7)	0.0301 (7)	0.0048 (6)	−0.0010 (6)	0.0001 (5)
C9	0.0177 (6)	0.0283 (7)	0.0253 (6)	0.0024 (5)	0.0045 (5)	0.0008 (5)
C10	0.0257 (9)	0.0216 (9)	0.0297 (9)	0	0.0002 (7)	0
C11	0.0196 (7)	0.0223 (6)	0.0180 (6)	−0.0001 (4)	0.0052 (5)	0.0006 (4)
C12	0.0234 (6)	0.0241 (7)	0.0219 (6)	−0.0010 (5)	0.0037 (5)	−0.0006 (5)
C13	0.0324 (8)	0.0225 (6)	0.0254 (6)	−0.0027 (5)	0.0051 (6)	−0.0004 (5)

### Geometric parameters (Å, °)

**Table:**

O1—C6	1.3612 (17)	C7—H7c	0.96
O1—H1	0.827 (17)	C8—H8a	0.96
N1—C9	1.4694 (15)	C8—H8b	0.96
N1—C10	1.4746 (10)	C8—H8c	0.96
N1—C11	1.4691 (15)	C9—H9a	0.96
C1—C2	1.4059 (19)	C9—H9b	0.96
C1—C6	1.4026 (19)	C10—H10	0.96
C1—C9	1.5211 (19)	C10—H10^i^	0.96
C2—C3	1.3945 (19)	C11—C11^i^	1.5132 (16)
C2—C7	1.508 (2)	C11—C12	1.5127 (17)
C3—C4	1.394 (2)	C11—H11	0.96
C4—C5	1.389 (2)	C12—C13	1.5376 (18)
C4—C8	1.503 (2)	C12—H12a	0.96
C5—C6	1.3923 (19)	C12—H12b	0.96
C5—H5	0.96	C13—C13^i^	1.5341 (18)
C7—H7a	0.96	C13—H13a	0.96
C7—H7b	0.96	C13—H13b	0.96
			
C6—O1—H1	106.8 (14)	H8b—C8—H8c	109.4716
C9—N1—C10	112.95 (8)	N1—C9—C1	111.10 (10)
C9—N1—C11	114.86 (9)	N1—C9—H9a	109.4708
C10—N1—C11	105.20 (7)	N1—C9—H9b	109.4711
C2—C1—C6	119.11 (12)	C1—C9—H9a	109.4716
C2—C1—C9	121.08 (12)	C1—C9—H9b	109.4713
C6—C1—C9	119.77 (12)	H9a—C9—H9b	107.791
C1—C2—C3	118.31 (12)	N1—C10—N1^i^	105.41 (8)
C1—C2—C7	121.47 (12)	N1—C10—H10	109.4714
C3—C2—C7	120.20 (12)	N1—C10—H10^i^	109.471
C2—C3—C4	123.46 (13)	N1^i^—C10—H10	109.471
C3—C4—C5	117.04 (12)	N1^i^—C10—H10^i^	109.4714
C3—C4—C8	123.25 (13)	H10—C10—H10^i^	113.2497
C5—C4—C8	119.71 (13)	N1—C11—C11^i^	100.00 (9)
C4—C5—C6	121.47 (13)	N1—C11—C12	116.90 (9)
C4—C5—H5	119.2656	N1—C11—H11	111.8254
C6—C5—H5	119.2654	C11^i^—C11—C12	111.59 (10)
O1—C6—C1	122.83 (12)	C11^i^—C11—H11	117.1086
O1—C6—C5	116.63 (12)	C12—C11—H11	100.2818
C1—C6—C5	120.53 (13)	C11—C12—C13	108.22 (10)
C2—C7—H7a	109.4708	C11—C12—H12a	109.4707
C2—C7—H7b	109.4713	C11—C12—H12b	109.4717
C2—C7—H7c	109.4707	C13—C12—H12a	109.4709
H7a—C7—H7b	109.4715	C13—C12—H12b	109.4717
H7a—C7—H7c	109.4716	H12a—C12—H12b	110.6913
H7b—C7—H7c	109.4714	C12—C13—C13^i^	113.08 (11)
C4—C8—H8a	109.4711	C12—C13—H13a	109.4717
C4—C8—H8b	109.4712	C12—C13—H13b	109.4711
C4—C8—H8c	109.471	C13^i^—C13—H13a	109.4708
H8a—C8—H8b	109.472	C13^i^—C13—H13b	109.472
H8a—C8—H8c	109.4704	H13a—C13—H13b	105.6049
			
?—?—?—?	?		

Symmetry codes: (i) −*x*+1, *y*, −*z*+3/2.

### Hydrogen-bond geometry (Å, °)

**Table:**

*D*—H···*A*	*D*—H	H···*A*	*D*···*A*	*D*—H···*A*
O1—H1···N1	0.827 (17)	1.880 (19)	2.6259 (13)	149.4 (19)
C12—H12B···O1^ii^	0.96	2.56	3.4998 (17)	166

Symmetry codes: (ii) −*x*+1, −*y*+1, −*z*+2.
